# Training for equipment maintenance and repair

**Published:** 2010-09

**Authors:** Sam Powdrill, Ismael Cordero, V Srinivasan

**Affiliations:** Assistant Professor, University of Kentucky College of Health Sciences, I Division of Physician Assistant Studies, 900 S Limestone Street, Lexington, KY 40536, USA.; Senior Clinical Engineer, ORBIS International, 520 8th Ave, llth Floor, | New York, NY 10018, USA.; Aravind Eye Care System, Madurai, India. Email: v.srinivasan@aravind.org

**Figure:**
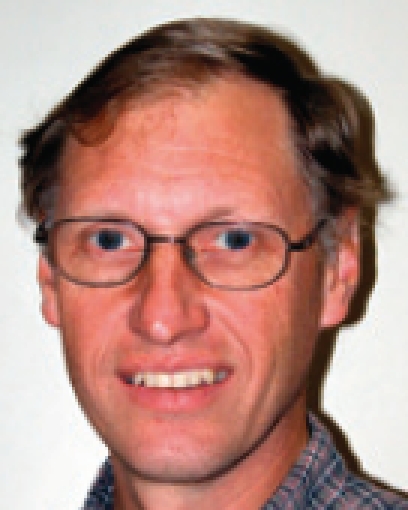


**Figure:**
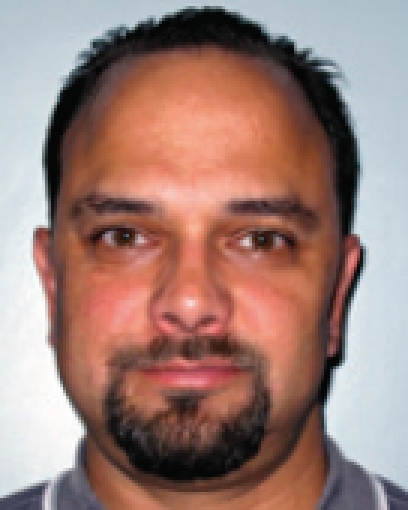


**Figure:**
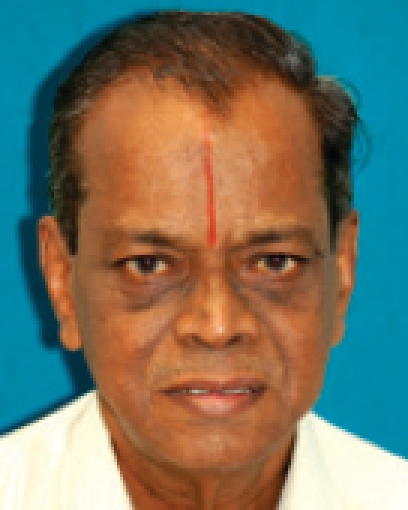


In order to ensure the that equipment functions well, both equipment users and the equipment maintenance and repair team must be trained. Users must be trained in basic care and maintenance of equipment, and the equipment team must be trained to undertake repairs and more complex maintenance tasks.

## Training equipment users

The primary responsibility for the care and maintenance of equipment rests with the user. Users should understand how their equipment works, what its limitations are, and what it can and cannot do. All of these are usually well described in the user manual that is supplied by the equipment manufacturer or supplier. It is important that users read and understand the user manual and keep it in a safe place.

In situations where surgeons or clinicians work under extreme time pressure, they may not be able to pay sufficient attention to the care and maintenance of the equipment they use. In this case, nursing staff and patient attendants can be trained in basic preventative maintenance and care. However, surgeons and clinicians must still be trained to use equipment properly and safely; they are also responsible for reporting faults and should be included in discussions about maintenance and repair.

Users should be trained to do the following **preventative maintenance** tasks on a regular basis (check the manufacturer's guide or user manual for details):

- Clean outer as well as inner surfaces and lubricated parts.
- Check for damage, loose or missing screws, and corrosion.
- Change filters and renewable parts.
- Lubricate movable parts.

All users, including clinicians, are responsible for the **safety** of their equipment. Users should be trained and encouraged to do the following:

- Carefully wipe the surfaces of the instrument regularly with a disinfectant, particularly those parts that come into contact with the patient, such as the chin rests on the slit lamp and keratometer.
- Check for sharp metal or broken lenses in the instrument that could injure the patient or user.
- Keep equipment, tubing, tools, and electrical cords out of the path of patients who may be blind and could trip over them.

### Top tips for training equipment users

- Demonstrate what to do.
- Allow the student to actually do the work and practice under supervision.
- Maintain a friendly environment, rather than a highly competitive environment, in which to learn.
- Be patient with your students, but expect effort and excellence.
- Always have a back-up plan in case equipment breaks or a part is not available.

**Figure:**
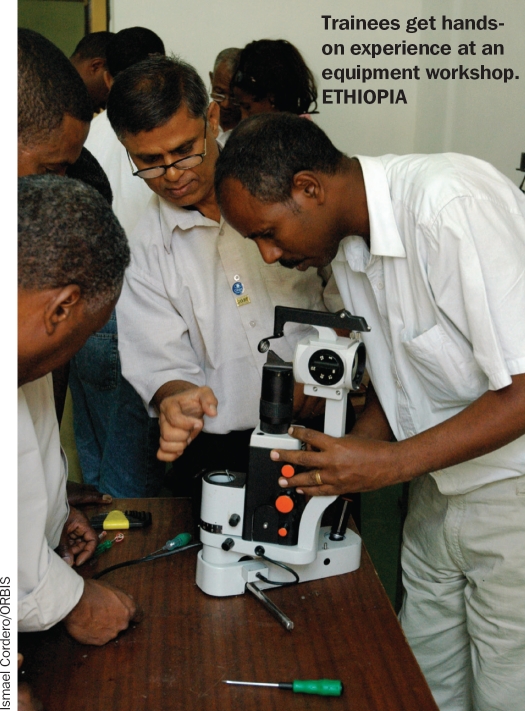
Trainees get hands-on experience at an equipment workshop. ETHIOPIA

Ismael Cordero/ORBIS

The person responsible for equipment in the eye unit (the ‘equipment person’) should periodically remind staff about the proper care and use of equipment, using the user manual as a guide to discussion.

## Training the equipment maintenance and repair team

Since new makes and models of equipment are constantly becoming available, the equipment maintenance and repair team needs to update its skills continually. Training should cover:

- Preventative maintenance and repair for maintainers
- Teaching preventative maintenance to users
- Maintenance management
- Management of stocks and stores
- Procurement procedures
- Financial planning and accounting
- How to work in a health facility environment.

The equipment team also needs other skills common to equipment users, such as:

- Basic do's and don'ts when handling equipment
- How to operate equipment
- Basic anatomy, physiology, and medical terminology
  Cleaning of equipment
- Safety procedures.

Training is not an activity that only happens once. Training is required at various times throughout an employee's career:

- Induction training: when staff are newly placed in post, move to a new department or facility, or to a new location with different responsibilities
- Training when new equipment first arrives
- Refresher training: regular training to update and renew skills throughout the working life of staff.

After training, the team can be expected to do the following:

- Communicate effectively with clinical personnel on medical equipment and safety issues.
- Train users to operate and care for equipment properly.
- Perform repairs in a cost-effective and timely fashion.
- Help to establish a safe environment for patients and staff.
- Take part in decision making about medical equipment management, planning, and procurement.

**TIP:** If an item of equipment is used far away from the location of the manufacturer or supplier and service personnel are not available, an effort must be made to obtain the service manual. This manual contains more detailed information than the user manual and is usually reserved for the use of contracted service personnel. Reading and understanding the service manual will give in-house maintainers the information they need. Keeping it safe is essential.

### Top training tips

- Send staff to factories that manufacture equipment.
- Invite engineers from manufacturers to visit your facility to conduct training on their equipment.
- Send staff to other locations which have already developed the skills required.
- Link the provision of training by the vendor to the procurement process.
- Run in-house (on-the-job) training sessions.
- Make use of regular clinical/professional meetings.
- Make use of academic courses at various levels.
- Approach local colleges to develop, run, and accredit new modules specifically designed for your equipment needs.
- Provide opportunities for practical, on-the-job experience.
- Provide opportunities for studying and teaching.
- Let maintenance staff attend peer group meetings or conferences.
- Provide various training materials for staff to refer to.
- Provide work placements (internships) for students in your workshop.

### Managing, motivating and retaining skilled staff

- Create multidisciplinary teams so that staff are not overstretched.
- Use suitable reporting and feedback methods so that staff know what is going on.
- Evaluate staff performance so that career development goals can be set.
- Help staff to develop their skills.
- Put in place suitable employment conditions such as a salary, holiday and sickness leave, and overtime entitlements.
- Ensure suitable working conditions, such as supportive supervision and suitable tools.

**Establishing and running an equipment workshop**

In general, hospitals with fewer than a hundred beds are more likely to save money and maintain quality by outsourcing equipment maintenance as opposed to having an in-house maintenance department. Most small health organisations simply cannot provide the needed resources, such as salaries for qualified technicians, to operate a good quality in-house workshop. However, larger hospitals may find it helpful to have their own workshop. The main benefits are:

- Better control over the maintenance budget
- Faster response speed
- Better understanding of user needs and organisational priorities.

You can find out whether an in-house equipment workshop will save costs: compare the money spent on maintenance performed by outside vendors to the anticipated initial investment and recurring expenses needed to establish and operate an in-house workshop. It is important to note that, even with an in-house workshop, there will always be a need for outsourced maintenance services, for example when the equipment is too complex for the in-house technicians or when repairs require special tools, test equipment, and service manuals. Most medium-sized health organisations will therefore have a mix of in-house and outsourced maintenance services.

In smaller hospitals, the role of medical equipment maintenance may be incorporated into the facilities maintenance department. Smaller hospitals that are part of a larger hospital system may also receive their medical equipment maintenance services from the medical equipment maintenance department of the central tertiary hospital of the system.

**What do you need?**

The workshop should be staffed by **maintenance personnel** of varying skill levels (artisans, technicians, and engineers) according to the amount and complexity of equipment in the health unit. As a rule of thumb, for every 100 beds at a district hospital there should be two medical equipment maintenance technicians.

**Figure:**
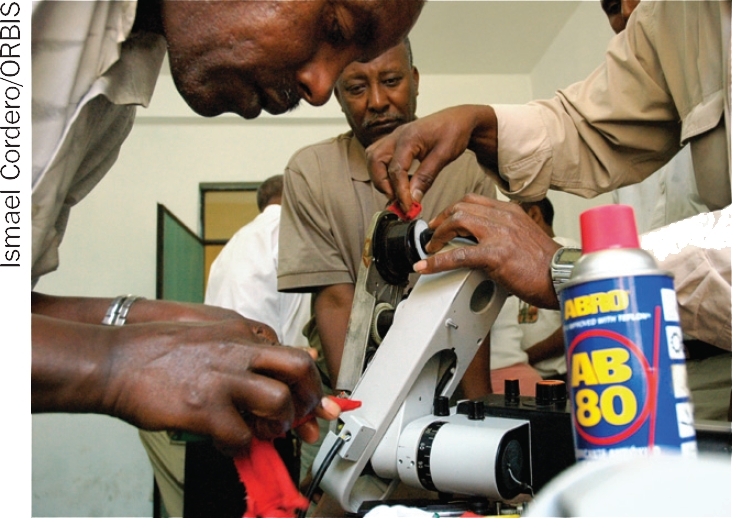
An equipment maintenance workshop in a district-level hospital. ETHIOPIA

Ismael Cordero/ORBIS

You will also need a **budget** to pay the setup and ongoing costs. The **setup costs** include the cost of tools, equipment, parts, materials, and the physical space for the workshop, as well as the costs of recruiting and training staff. The **ongoing costs** are salaries, consumables, spare parts, replacement tools, and ongoing training.

It is important to have an **equipment workshop management plan** that includes department policies, procedures, standards, and guidelines.

The **workspace** must be big enough to accommodate the equipment technicians and their physical resources. Maintenance work on eye equipment, in particular, requires a separate workspace that can be kept clean to avoid damage to lenses, etc. You will also need:

- Workbenches, stools, shelves and other furniture
- An office area with desks, filing cabinets, a notice board, telephone, etc.
- Work lights
- Repair tools
- Test and calibration equipment
- Safe storage for user and service manuals
- Sufficient number of electrical outlets
- Ventilation
- Running water and a sink
- Secure storerooms for spare parts and materials
- Secure outside storage areas for gas bottles, old or unrepairable equipment awaiting safe disposal, etc.
- Where possible, a computer for keeping your equipment inventory and repair records and accessing the internet to obtain technical information, source vendors and parts, and participate in equipment maintenance discussion groups to solve problems.

You should have enough **spare parts** in stock, which may need to be pre-ordered from the manufacturer or distributor. Useful spare parts to have include specialised light bulbs, gaskets, air filters, and other equipment-specific parts that wear out frequently.

Most of the other maintenance materials you need can be found in local markets, such as oil, grease, electric cables, washers, screws, fuses, generic light bulbs, cleaning agents, disinfectant solutions, brushes, and cloths.

